# Efficient Multicriteria Protein Structure Comparison on Modern Processor Architectures

**DOI:** 10.1155/2015/563674

**Published:** 2015-10-28

**Authors:** Anuj Sharma, Elias S. Manolakos

**Affiliations:** Department of Informatics and Telecommunications, University of Athens, Athens, Greece

## Abstract

Fast increasing computational demand for all-to-all protein structures comparison (PSC) is a result of three confounding factors: rapidly expanding structural proteomics databases, high computational complexity of pairwise protein comparison algorithms, and the trend in the domain towards using multiple criteria for protein structures comparison (MCPSC) and combining results. We have developed a software framework that exploits many-core and multicore CPUs to implement efficient parallel MCPSC in modern processors based on three popular PSC methods, namely, TMalign, CE, and USM. We evaluate and compare the performance and efficiency of the two parallel MCPSC implementations using Intel's experimental many-core Single-Chip Cloud Computer (SCC) as well as Intel's Core i7 multicore processor. We show that the 48-core SCC is more efficient than the latest generation Core i7, achieving a speedup factor of 42 (efficiency of 0.9), making many-core processors an exciting emerging technology for large-scale structural proteomics. We compare and contrast the performance of the two processors on several datasets and also show that MCPSC outperforms its component methods in grouping related domains, achieving a high *F*-measure of 0.91 on the benchmark CK34 dataset. The software implementation for protein structure comparison using the three methods and combined MCPSC, along with the developed underlying *rckskel* algorithmic skeletons library, is available via GitHub.

## 1. Introduction

Proteins are polypeptide chains that take complex shapes in three-dimensional space. It is known that there is a strong correlation between the structure of a protein and its function [1]. Moreover, beyond evolutionary relationships encoded in the sequence, proteins' structure presents evidence of homology even in sequentially divergent proteins. Comparison of the structure of a given (query) protein with that of many other proteins in a large database is a common task in Structural Bioinformatics [2]. Its objective is to retrieve proteins, with those of similar structure to the query being ranked higher in the results list.

*Protein Structure Comparison* (PSC) is critical in homology detection, drug design [3], and structure modeling [4]. In [5–7] the authors list several PSC methods varying in terms of the algorithms and similarity metrics used, yielding different, but biologically relevant results. There is currently no agreement on a single method that is superior for protein structures comparison [8–13]. Hence, the modern trend in the field is to perform* Multicriteria Protein Structure Comparison* (MCPSC), that is, to integrate several PSC methods into one application and provide consensus results [14]. The approach banks on the idea that an ensemble of classifiers can yield better performance than any one of the constituent classifiers alone [15–17].

Pressing demand for computing power in the domain of protein structures comparison is the result of three factors: ever increasing size of structure databases [18], high computational complexity of PSC operations [19], and the trend towards applying multiple criteria PSC at a larger scale. So far this demand has been met using distributed computing platforms, such as clusters of workstations (COWs) and computer grids [8, 14, 20]. While distributed computing is popularly used in PSC, the parallel processing capabilities of modern and emerging processor architectures, such as Graphics Processing Units (GPUs), multi- and many-core Central Processing Units (CPUs) have not been tapped. These parallel processing architectures have become more readily available [21, 22] and instances of their use are beginning to appear in the broader field of biocomputing [23, 24]. These processor architectures can in principle be used additively to meet the ever increasing computational demands of MCPSC by complementing already in use distributed computing approaches [25].

Multicore and many-core CPUs, as opposed to GPUs, retain backward compatibility to well-established programming models [26] and offer the key advantage of using programming methods, languages, and tools familiar to most programmers. Many-core processors differ from their multicore counterparts primarily on the communication subsystem. Many-core CPUs use a Network-on-Chip (NoC) while multicore CPUs use bus-based structures [27].* The Single-Chip Cloud Computer* (SCC) experimental processor [28] is a 48-core NoC-based concept-vehicle created by Intel Labs in 2009 as a platform for many-core software research. While multicore CPUs are ubiquitous and easily available for parallel processing, due to the drive from leading chip manufacturers over the past several years [29], many-core CPUs are not as deeply entrenched or commonly available yet. However, due to architectural improvements many-core processors have the potential to deliver scalable performance by exploiting a larger number of cores at a lower cost of intercore communication [30, 31]. Moreover, many-core processors offer improved power efficiency (performance per watt) due to the use of more “light-weight” cores [26, 32]. All these factors make modern CPUs (multicore and many-core) powerful technologies suitable for meeting the high computational demands of MCPSC computations by employing scalable parallel processing. Both multicore and many-core processors have been used in bioinformatics [33] mainly for pairwise or multiple sequence comparison [34, 35]. However, NoC architectures have not yet been extensively employed despite the flexibility and parallel processing capabilities they offer [35].

In this work, we extend the framework presented in [36] for MCPSC. We augment the software with implementations of two popularly used PSC methods—*Combinatorial Extension* (CE) [37] and* Universal Similarity Metric* (USM) [38]—in addition to* TMalign* [39], and then combine them to create a scalable MCPSC application for the SCC. We also develop an equivalent MCPSC software implementation for a modern multicore CPU. We combine sequential processing (for USM PSC tasks) and distributed processing (for TMalign and CE PSC tasks), to obtain high speedup on a single chip. Our solution highlights the flexibility offered by these architectures in combining the parallel processing elements based on the requirements of the problem. We analyze the characteristics of the MCPSC software on the SCC with a view of achieving high speedup (efficiency) and compare the performance to that of the multicore implementation. Contrasting the performance of multicore and many-core processors, using multiple datasets of different sizes, highlights the advantages and disadvantages of each architecture as applicable to MCPSC. To the best of our knowledge, this is the first attempt in the literature to develop and compare MCPSC implementations for multicore and many-core CPUs.

## 2. Methods

### 2.1. Experimental Systems Used

**Figure 1:**
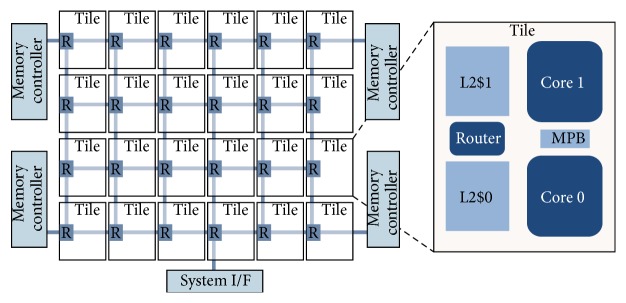
The Intel Single-Chip Cloud Computer. It is a network on chip processor architecture having a mesh of 4 by 6 = 24 routers (R) connecting the 24 “tiles” with 2 cores per tile [28].

The Single-Chip Cloud Computer (SCC) is an experimental Intel microprocessor architecture containing 48 cores integrated on a silicon CPU chip. It has multiple dual ×86 core “tiles” arranged in a 4 × 6 mesh, memory controllers, and a 24-router mesh network as depicted in Figure 1. Although it is a “concept-vehicle” [40] the technology it uses is scalable to more than 100 cores on a single chip [28]. The SCC resembles a cluster of computer nodes capable of communicating with each other in much the same way as a cluster of independent machines. Salient features of the SCC hardware architecture relevant to programming the chip are listed in Table 1.

Further, each core of the SCC has L1 and L2 caches. In the SCC architecture, the L1 caches (16 KB each) are on the core while the L2 caches (256 KB each) are on the tile next to the core with each tile carrying 2 cores. Further, each tile also has a small message passing buffer (MPB), of 16 KB, and is shared among all the cores on the chip. Hence, with 24 tiles, the SCC provides a Message Passing Buffer Type (MPBT) memory of size 384 KB. The SCC therefore provides a hierarchy of memories usable by application programs for different purposes including processing and communication. A reserved bit in the page table of the cores is used to mark MPBT data. Legacy software by default does not mark memory as MPBT and runs without modifications. If the bit is set, data is cached in L1 and bypasses L2.

To benchmark performance we run all-to-all PSC using different methods on a single and on multiple cores of the SCC. Each core is a P54C Intel Pentium (32-bit) running SCC Linux. We have also used a PC, with a 3 GHz AMD Athlon II X2 250 64-bit processor and 3 GB RAM running Debian Sid, to analyze the characteristics of individual PSC methods and develop PSC job partitioning schemes. Lastly, a multicore baseline was established by running all-to-all MCPSC, using the multithreaded version of MCPSC on a Intel Core i7-4771 “Haswel” Quad-Core CPU running at 3.5 GHz with 8 GB of RAM and a SSD running Debian Sid. The Core i7 CPU features highly optimized out-of-order execution and HT (Hyper Threading) [41], Intels flavor of Simultaneous Multithreading (SMT). All software developed was compiled with the GNU C Compiler version 4.8.

### 2.2. Software Framework for Implementing PSC Methods

We introduced in [36] a framework for porting a PSC method to the SCC and have used it to implement a master-slaves variant of the TMalign PSC method [39]. The framework allows developing efficient parallel implementations using basic functionality provided by our* rckskel* skeletons library for a many-core processor. Algorithmic skeletons allow sequential implementations to be parallelized easily without specifying architecture dependent details [42]. By nesting and combining skeletons the desired level of parallelism can be introduced into different PSC methods. While several algorithmic skeleton libraries have been developed [42, 43], none of them has been targeted specifically for many-core processor architectures. Using* rckskel* allowed efficient usage of the SCC cores to speed up all-to-all PSC. In this work, we extend the framework to handle MCPSC while allowing the methods to be combined serially where required.

In Algorithm 1 we provide the pseudo-code of the Multicriteria PSC application for the general case of comparing every element in a set of query proteins *Q* with every element in a set of a reference database of proteins *D* using three PSC methods. It covers both the “all-to-all” comparison, where the set of query proteins is the same as the set of database proteins, and the “many-to-many” comparison, where the query proteins are different from the database proteins. We used the MCPSC application developed to measure completion times of all-to-all PSC. The size of the job list generated for parallel processing consists of *K*_*Q*_ × *K*_*D*_ × *M* PSC jobs, where *K*_*Q*_ (*K*_*D*_) is the number of proteins in set *Q* (*D*), respectively, and *M* is the number of PSC methods implemented using parallel processing (*M* = 2 currently). This is the most fine grained job distribution that can be obtained without parallelizing the pairwise PSC operation.

The* rckskel* library [36] was upgraded in this work to C++ allowing for more flexibility in usage. The choice of the programming language was motivated by two requirements: seamless handling of communication data and type safety. In order to perform parallel operations data must be passed between the cores of the SCC. In addition to the control structures built into the library this includes user data. Since the user data can vary depending on the user application, the library needs to provide support for any data structures to be passed between the cores. This was achieved by using the boost object serialization/deserialization construct, which allows the library to accept any arbitrary data type from the user as long as it can be serialized. By making use of the C++ template extension the skeletons in* rckskel* are completely type safe. This implies that any inconsistency in the data flow described by the user while constructing the skeleton hierarchy is caught at compile time instead of run time, which significantly simplifies developing complex parallel programs using the library.

### 2.3. MCPSC Application Development for Many-Core Processor

In order to perform Multicriteria PSC an application was developed using our software framework incorporating three PSC methods. For the CE and USM methods we ported existing software to the SCC. The implementation of CE builds on existing C++ sources which require a small structure manipulation library as an external binary. This library was modified in order to link it to the main CE code and deprecate the need for the program to run as an external binary. Further, the code was modified to compare only the first chains of the domains as in TMalign. We then generated a master-slaves parallel implementation of the C/C++ code, using* rckskel*, similar to the master-slaves port we have developed for TMalign in [36]. We also developed a C++ implementation of USM, based on the existing Java sources [38], so that we could integrate it with the rest of the software. As per the existing implementation, the Kolmogorov complexity of the proteins was approximated with the* gzip* of their contact maps. Therefore, we take it that the normalized compression distance between the contact maps of a pair of proteins expresses their USM similarity. In the final implementation, the master process executes the USM jobs sequentially, due to the fast processing times for these jobs. Subsequently, the master distributes the CE and TMalign PSC jobs to the available slaves to be executed in parallel on different cores. Moreover, we extended significantly the previous implementation [36] by adding load balancing. The software framework supports both dynamic round-robin [44] and static jobs partitioning, thus allowing experimentation with different methods and comparison in order to select the best approach for the given many-core processor and dataset.

### 2.4. MCPSC Application Development for Multicore Processor

In addition to implementing the MCPSC framework for the SCC we also developed an implementation for multicore processors. Intercore communication calls handled in the NoC implementation by* rckskel* are replaced by reference passing between multiple-threads implemented using OpenMP. The resulting software combines the three methods, USM, CE, and TMalign, in the same way as the NoC implementation. The USM jobs are, therefore, processed serially while CE and TMalign are processed in parallel. Since the three methods are combined into one executable, the structure data for each protein domain is loaded only once and reused whenever the domain appears in a pairwise comparison being performed. This reduces the overall processing time as compared to running each method currently available software, where only pairs are accepted thereby requiring multiple reloads of each protein domain to perform all-to-all analysis (*N* + 1 times for each domain if the dataset contains *N* domains). Further, the software allows direct comparison between the performance of many-core and multicore processors for the MCPSC problem because all other factors, algorithms and data, remain constant.

### 2.5. MCPSC Consensus Score Calculation

In both implementations, for each protein pair, a total of *M* comparison scores are returned, where *M* is the number of PSC methods employed. These results are stored in a *P* × *M* matrix, where *P* is the total number of protein pairs in a dataset. Individual method comparison scores may, or may not, be normalized, depending on the PSC method used; therefore, we normalize all scores in the matrix between 0 and 1 along each column (feature scaling [45]) that is along each PSM method used. The normalization process proceeds as follows. First the minimum and maximum values for each column of the matrix, containing the dissimilarity values, are determined. Then, the matrix is scaled by dividing the difference of column elements and the column minimum value by the difference of the column maximum and minimum value as shown in (1) (where *i* is the row index and *j* is the column index). Note that the minimum value of any column cannot be less than zero because these are PSC scores with a minimum value of zero. Once the columns have been normalized, a consensus MCPSC score is calculated for each protein pair by taking the average of the *M* values corresponding to that protein pair. It must be noted that the smaller the pairwise PSC score the higher the similarity between the pair proteins:

(1) $$\begin{array}{c} X_{i,j}^{\prime }=\frac{X_{i,j}-\min\left(X_{j}\right)}{\max\left(X_{j}\right)-\min\left(X_{j}\right)}. \end{array}$$

### 2.6. Datasets Used

Several datasets, listed in Table 2, were used in this work. The table includes statistics about the length of the protein domains and the distribution of the SCOP [46] classifications in each dataset. It can be seen that there is significant variation in the sizes of the datasets, the lengths of protein domains they contain, and the distribution of the SCOP classifications. For each dataset we retained domains where the PDB file could be downloaded and a classification could be found for the domain in SCOP v1.75. Further, the scripts available for download with the* USM* sources were used to extract the contact maps from the PDB files. The Chew-Kedem (CK34) and the Rost-Sander (RS119) datasets were used to develop the load balancing schemes for the SCC and to study the speedup (throughput) on the i7 processor. Further, all the datasets were used to compare the performance of the SCC with that of the i7 on all-to-all MCPCS processing.

### 2.7. *F*-Measure Analysis for the Chew-Kedem Dataset

We obtained all-against-all comparison scores for all comparison methods, and based on these scores we performed pairwise hierarchical clustering for each method, using* hclust* from the stats package in *R* [47]. We calculated the *F*-measure of the hierarchical clustering for each PSC methods (TMalign, USM, CE, and MCPSC). The *F*-measure is calculated using (2), where *C* is the number of clusters (*C* = 5 in this work) as described in [48]

(2) True Positives (TP)=∑i=1Cnumber of correctly assigned domains,False Positives (FP)=∑i=1Cnumber of incorrectly assigned domains,False Negatives (FN)=∑i=1Cnumber of missing domains,Precision  P=TPTP+FP,Recall  R=TPTP+FN,F-measure  F=2∗P∗RP+R.

## 3. Results and Discussion

### 3.1. Baseline All-to-All PSC

Table 3 provides the times taken to perform all-to-all comparison on the PC and on a single core of the SCC, with the CK34 and RS119 datasets. The software took longer to load data and process jobs on a single core of the SCC as compared to the PC. The longer data loading times are due to the need for network I/O, since the data is stored on the Management Console PC (MCPC) and accessed by the SCC via NFS. In long running services, however, data is loaded once and used when needed; therefore, this time is disregarded in our performance comparisons. The differences in processing times are due to the architecture difference of the processor cores (×86-64 versus ×86) and their operating frequencies (3 GHz versus 533 MHz) on the PC and a single SCC core, respectively.

We performed a statistical analysis, using SecStAnT [49], of the proteins participating in the 10 slowest and the 10 fastest pairwise PSC tasks (using TMalign and CE) using the CK34 and RS119 datasets. The parameters on which SecStAnT compares proteins are their secondary structure characteristics: Bond Angle, Dihedral Angle, Theta, and Psi. Results of the 1-parameter and 2-parameter statistical analysis, distributions, and correlations did not show any significant statistical difference between the slowest and fastest PSC domains. However, the slowest pairs contain the Hlx310Alpha supersecondary structure which is absent from the fastest pairs. The 310-helix occurs rarely in protein domains, because the tight packing of the backbone atoms makes it energetically unstable. This structure might be responsible for the increased alignment times in certain domains. Further, we compared the proteins using their UniPort entries and observed that most proteins in the slowest set are Cytoplasmic, while most members in the fastest set are membrane proteins.

### 3.2. Relation of Expected PSC Times to the Lengths of the Proteins under Comparison

**Figure 2:**
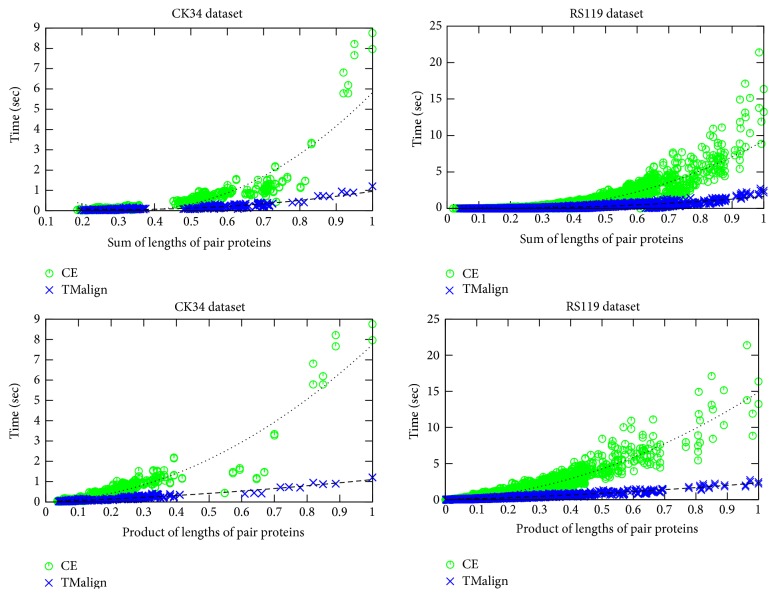
Comparison of pairwise PSC processing times using TMalign and CE, with respect to the normalized sum of lengths and normalized product of lengths of pair proteins. The dotted lines show the quadratic best fit for the data.

Comparison of the pairwise PSC processing with respect to the protein lengths, as shown in Figure 2, reveals some interesting facts: the time required for completing a pairwise PSC task is clearly a function of the lengths of the proteins forming the pair. USM requires extremely small pairwise comparison times, as shown in Table 1, and was thus excluded from this analysis. The best fit is achieved when using the product of lengths of the pair proteins as an attribute and is better than the fit achieved when using the sum of the lengths. Moreover, the quadratic curve fitting results are better than the linear as shown in Table 4. For a given PSC method the relative times required for completing a pairwise PSC task depend on the properties of the pairs participating. However, the absolute time required for completing a pairwise PSC task depends also on the complexity of the method used. Since the complexity of one pairwise PSC method as compared to another cannot be known a priori, it cannot be used as a factor for a generic jobs partitioning scheme for load balancing purposes.

### 3.3. Speedup and Efficiency Analysis of Multiple Criteria PSC on the SCC NoC CPU

In this work, we experimented with both round-robin and job partitioning schemes for load balancing. Given the close correlation between lengths of pair proteins and processing times we used this as criteria for both. Round-robin [44] is a dynamic load balancing method in which the master process maintains a list of jobs and hands the next job in the list to the next free slave process. It is worth noting that if the list is presorted based on attributes that are known to be proportional to the processing times this would naturally result in little to no idle times for the slave processes. For static job partitioning, determining job-to-core mapping is equivalent to constructing *N* equal partitions for a list of elements, which is an NP-hard problem [50]. In our case *N* is the number of cores and is equal to 47 when all SCC cores are used. We investigated methods for PSC jobs partitioning based on the sum and the product of the lengths of the two proteins to be compared. We have statically partitioned the PSC jobs using as attribute the sum of lengths (product of lengths) of the paired proteins to be compared. The partitions are generated using the Longest Processing Time (LPT) algorithm: the jobs are sorted in descending order by sum of lengths (product of lengths) and then assigned to the partition with the smallest total running sum. These partitions will be referred to as “Greedy” partitions from now on. Since the job partitions are processed on different cores this strategy is expected to balance the processing times by reducing idle times.

**Figure 3:**
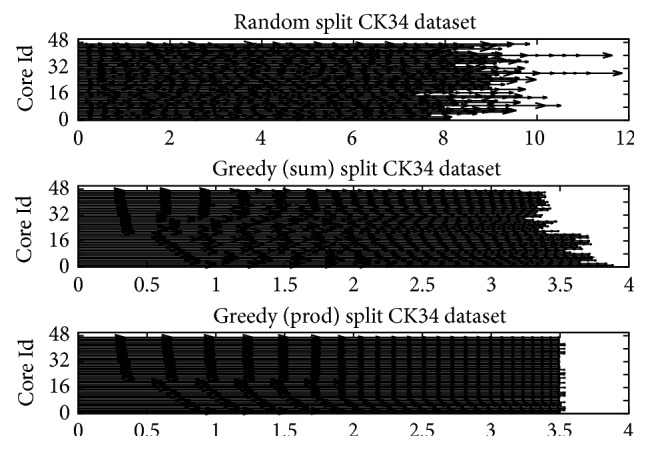
The 47 partitions created from list of PSC tasks sorted randomly or by the sum (product) of lengths of pair proteins. Each horizontal line represents the sum of the normalized lengths of the protein pairs assigned to that partition.

Figure 3 shows the partitions generated using a random partitioning and a greedy partitioning scheme based on the sum of lengths and the product of lengths as attributes. Partitions created with the random scheme, with equal number of pair proteins per partition, vary greatly in terms of the total normalized sum of the sum (or product) of the pair protein lengths. The size of this normalized sum is expected to be proportional to the overall processing time each partition will require when assigned to a core. Therefore, if each partition of PSC tasks created with the random scheme is processed on a different core, several cores will have significant idle times at the end. On the contrary, partitions created with the greedy scheme are almost equal as to the normalized sum of the sum (or product) of the pair protein lengths. Hence, if each partition of PSC tasks created by the greedy scheme is processed on a different processing element (PE), idle times are expected to be minimized.

We extended the framework introduced in [36] by incorporating more PSC methods (CE and USM) in addition to TMalign and computing consensus scores in order to perform Multicriteria PSC. We retain the master-slaves processing model in the extended framework; the first available core runs the master process and all other cores run slave processes. The master loads the data and generates a list of jobs, each one involving a single pairwise Protein Structure Comparison operation to be performed using a specified PSC method. Due to the vastly different processing times of PSC operations when using different methods, we allow the master process: (a) to make a choice between sequential versus parallel job processing and (b) implement a load balancing scheme for the slaves. The first allows the master to sequentially process jobs of a PSC method when distributing them which would actually result in an increased processing time. The second allows the master to reduce idle times in slave processes which perform PSC continuously, receiving jobs from and returning the results to the master, till they receive a terminate signal.

The consensus value for a protein pair is calculated using the scores from the three PSC methods: (a) the* USM-metric* from USM, (b) the* TM-score* from TMalign, and (c) the* Root Mean Square Distance* (RMSD) from CE. The USM-metric and RMSD are distances and have a zero value for identical structures. The TM-score is therefore inverted since it is the only one of the three which is a similarity metric. At the end the average of the three normalized dissimilarity scores is taken as the MCPSC score for the protein pair.

In Table 5 we compare the performance of the master-slaves setup, with one master and 47 slaves, to that of a single core of the SCC and show the speedup and the efficiency achieved using the CK34 and RS119 datasets. TMalign and CE jobs were run under the master-slaves model, while USM jobs were processed by the master due to the negligible time required by these jobs.

The greedy static partitioning scheme improves on the performance of the random partitioning scheme, but it is inadequate for the SCC where the cost of data transfer between cores is low. This scheme would be of interest in clusters of workstations where interconnection networks are slow. The greedy partitioning scheme could also be beneficial on larger many-core processors, where the master may become a bottleneck, making off-line creation of batches of PSC tasks essential. In such a scenario however, other techniques, such as work-stealing, would need to be assessed before selecting the most appropriate method.

The round-robin dynamic job assignment strategy outperforms the best offline partitioning scheme. Furthermore, the sorted round-robin flawlessly balances out the cores processing times on both datasets, making it the best approach for distributing jobs to the cores of the SCC. In this approach, the protein pairs are presorted in descending order of product (or sum) of pair protein lengths. Specifically, for the one-to-many PSC case, we simply retrieve the database proteins in descending order of length and create pairwise PSC tasks with the query protein. For the many-to-many PSC case however, we need to determine the correct order of the proteins when the query proteins are received.

The sorted round-robin strategy distributes the jobs most efficiently for the proposed master-slaves setup on the Intel SCC with 47 slaves. This setup achieves a 42-fold (40-fold) speedup for the RS119 (CK34) dataset as compared to a single core of the SCC. The speedup is almost linear, which suggests that high efficiency can be achieved even when the many-core processor has more nodes. A bigger many-core processor, however, may require using a hierarchy of masters to avoid a bottleneck on a single master node. In such a scenario, a combination of partitioning schemes and round-robin job assignment could be used to distribute and minimize idle times. For instance, on larger NoC processors a well-balanced solution may require the use of clusters of processing elements, concurrently processing PSC jobs with different methods. All PEs computing jobs of a PSC method would receive jobs from a specific submaster. All such submasters would in turn receive jobs from a common global master.

### 3.4. Comparison of Multicriteria PSC Performance on Multicore and Many-Core CPUs

In order to perform this experiment the MCPSC software framework was retargeted to the Intel Core i7 multicore processor, using* openmp* [51] to implement multithreading. This experiment allowed us to assess how the MCPSC problem scales with increasing number of cores on a modern multicore CPU readily available for scientists and engineers. This multicore software version uses shared memory to replace the RCCE based message passing (*recv* and* send*) calls used in the many-core version developed for the Intel SCC. As shown in Table 6 we observe speedup on this multicore processor when running all-to-all MCPSC with the CK34 and RS119 datasets up to the 4 threads configurations. Thereafter a steep speedup drop (efficiency loss) is observed.

This is attributed to the fact that this is a quad-core processor that implements Hyper Threading (HT) [41]. Since all threads operate on exactly the same type of instructions workload (SPMD) it cannot take advantage of each core's super scalar architecture that needs varied workload placement (e.g., integer and floating point arithmetic at the same time) to show performance advantages when utilizing more threads than cores. Further, an SMP Operating System (O/S), like the one we run on our test system, uses all system cores as resources for swapping threads in and out. A thread executing on core *PE*_1_ for some number of cycles may get swapped out and later resume execution on core *PE*_2_. Thus, the thread cache on core *PE*_1_ becomes useless and it gets a lot of cache misses when restarting on *PE*_2_. This O/S behavior impacts cache performance and reduces the overall application performance. In terms of pairwise-PSC tasks per second (pps) the Intel Core i7 achieves a throughput of 13.47 pps (9.45 pps) on the CK34 (RS119) dataset as compared to 5.53 pps (3.63 pps) achieved by the SCC. This is due to the fact that the multicore CPU contains latest generation cores, featuring a highly superior, out-of-order microarchitecture and clocked at almost 7.5x the frequency of the SCC. We believe that even more performance can be exploited from next generation multicore processors, should they start introducing hardware memory structures like the Message Passing Buffer (MPB) of the SCC NoC processor alongside their cores, for increased communication efficiency among them, without resorting to shared memory and its unavoidable locks or cache-coherency protocol overheads.

Finally, Table 7 shows the comparative performance of the SCC (with 48 cores) and the i7 (running 7 threads) on all-to-all MCPSC using several datasets. It can be seen that the i7 outperforms the SCC consistently in terms of throughput. The two larger datasets, Lancia and Proteus, were not processed on the SCC because of the limited per-core memory (512 MB) which was not sufficient to load the domain structure data for the full dataset. A decrease in throughput was observed as the size of the dataset increases with some exceptions. Both the SCC and the i7 deliver lower than expected throughput (based on its size) on the Fischer dataset which we believe is due to the higher complexity of the dataset. As evidenced by Table 2 the Fischer dataset has domains belonging to 5 times more SCOP Super Families as compared to CK34 and also has the second highest median length of protein domains among all the datasets used in this work. Conversely, the throughput delivered by the i7 on the Lancia dataset is significantly higher than that delivered for the similar size (in terms of number of domains) Proteus dataset. We attribute this difference to the large difference between the mean lengths of domains in each dataset as noted in Table 2.

### 3.5. Qualitative Analysis of MCPSC Results Based on the Chew-Kedem Dataset

The acceleration capabilities offered by modern processors (many-core and multicore) enables performing all-to-all PSC using different methods on large-scale datasets and compare results. For example, it becomes easy to perform a qualitative analysis on a set of protein domains and use consensus score (GP'S) to explore relation between the protein domains, categorize them into biologically relevant clusters [48], and automate generation of databases such as* Structural Classification of Proteins* (SCOP) [46].

**Figure 4:**
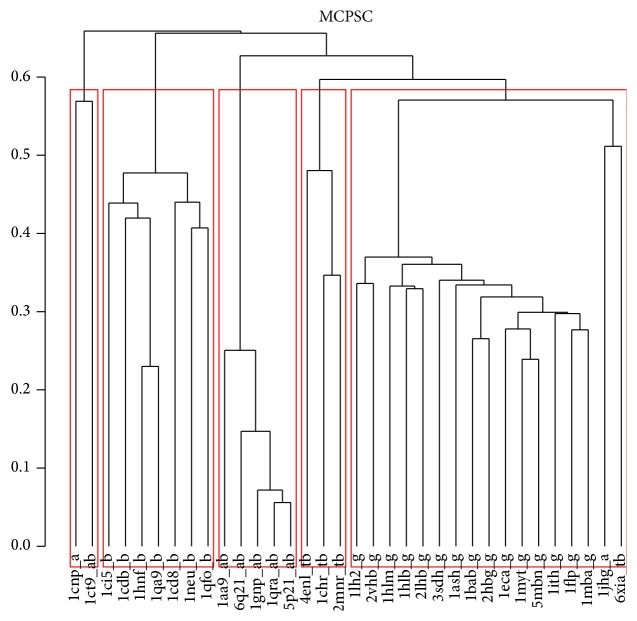
Hierarchical clustering result using the Chew-Kedem dataset and MCPSC score as distance metric between domains. Each box represents a cluster and the domains belonging to it. The average linkage method was used to build the dendrogram.

The MCPSC comparison method outperforms the component PSC methods (TMalign, CE, and USM) and groups 34 representative proteins from fivefold families into biologically significant clusters. In Figure 4 each protein domain is using the format “domainName_foldFamilies.” The tags of the “foldFamilies” belong to one of (i) tb: TIM barrel, (ii) g: globins, (iii) ab: alpha beta, (iv) b: all beta, and (v) a: all alpha protein families. The clusters obtained from the MCPSC method show that most domains are grouped correctly according to their structural fold. In comparison all the component PSC methods produce clusters in which there are many wrongly grouped domains. Details of domain cluster allocation of all four methods are included in the Supplementary Materials file (see Supplementary Material available online at http://dx.doi.org/10.1155/2015/563674)* dom_clust.xls* with clusters generated by USM containing the highest number of errors. The quantitative analysis evaluating the Recall versus Precision tradeoff based on the *F*-measure [52] results in a value of *F* = 0.91 for the MCPSC method, which is higher than TMalign (*F* = 0.82), CE (*F* = 0.71), and USM (*F* = 0.62). Finally in the Supplementary Material file* supplementary_material.pdf* we provide clusters generated by* hclust* [47], for the four PSC methods (see Figures S1, S2, and S3). PSC scores generated for all the datasets for all PSC methods are included in the Supplementary Material file* psc_scores.zip*.

## 4. Conclusions and Future Work

In the near future, we will see a continued increase in the amount of cores integrated on a single chip as well as increased availability of commodity many-core processors. This trend is already visible today in that off-the-shelf PCs contain processors with up to 8 cores, server grade machines often contain multiple processors with up to 32 cores, and multicore processors are even appearing in average grade mobile devices. Further, the success of GPUs, Tilera's architecture, and Intel's initiative for integrating more and more cores on a single chip also attest to this trend. The ubiquity and the advanced core architectures employed by multicore CPUs make it imperative to build software that can efficiently utilize their processing power. Our experiments show that a modern Core i7 is able to deliver high throughput for all-to-all MCPSC. Utilizing the performance gain delivered by multicore CPU becomes especially important when considered in combination with distributed setups, such as clusters, which may already contain nodes with multiple cores.

Both multicore and many-core architectures lend themselves to the popular and powerful MapReduce parallel programming model [53]. If the underlying communication libraries—typically built on top of Message Passing Interface (MPI) [54]—are available, this highly popular approach can be applied to large-scale MCPSC in a manner similar to its application in other domains of bioinformatics [55]. While some implementation of MapReduce is available for multicore machines, it must be noted that no MapReduce framework implementations are available for the SCC. One possible approach for using MapReduce in MCPSC would be to (a) determine pairwise PSC to be performed on each PE (pre-Map), (b) run all PSC methods on all pairs on each PE in parallel (Map), (c) collect PSC scores from all PEs to a designated PE (shuffle), and (d) combine and generate final MCPSC scores (Reduce). Movement of data for task distribution (pre-Map), intermediate data transfer performed by the “shuffle” mechanism, and collection of results if required by the Reduce step would benefit from low cost of inter-PE communication. Load balancing methods similar to those discussed in this work could be useful in determining effective strategies for the distribution of PSC tasks (pre-Map). Analysis of MCPSC performance on multicore and many-core processors with MapReduce would be required to understand the design trade-offs that would lead to efficient designs.

Scalability limitation of the bus-based architecture coupled with the greater potential of Network-on-Chip based processors to deliver efficiency and high performance implies that NoCs are likely to be used extensively in future many-core processors. It is therefore important to start developing software frameworks and solutions that capitalize on this parallel architecture to meet the increasing computational demands in structural proteomics and bioinformatics in general. Comparison of many-core and multicore processors in this work shows that the former can deliver higher efficiency than the latter processor architectures. Further, our experimental results make it clear that Intel's SCC NoC matches the speedup and efficiency achieved by a cluster of faster workstations [14]. However, a market ready NoC CPU will have a much better per watt performance as compared to a cluster while also saving in space and infrastructure management costs. Additionally any savings in watts consumed also reflect in savings in cooling infrastructure required for the hardware. Furthermore, with the per watt performance being in focus for processor manufacturers, such as Intel and Tilera, this gap is set to expand even further. Finally many-core processors when used as the CPUs of near-future clusters of workstations will provide an additional level of local parallelism to applications where performance scalability is essential to tackle ever increasing computational demands. As this study demonstrates, the likely increase in the availability and ubiquitousness of many-core processors, the near linear speedup in tackling the MCPSC scenario, and the ease of porting new PSC methods to NoC based processors make many-core processors be of great interest for the high performance structural proteomics and bioinformatics communities in general.

Incorporating additional PSC methods to a many-core NoC processor is straightforward due to the familiar programming model and architecture of the individual cores. Our* rckskel* library [36] offers convenience and parallel programming functions to further accelerate development on the NoC. On future larger NoC processors optimal solutions may require the use of clusters of cores, concurrently processing PSC jobs with different methods. Using the* rckskel* library would facilitate the software development, for such more complicated scenarios, hiding low level details from the users. The advantages offered by an algorithmic skeletons library like the* rckskel* make it of interest to further develop the library. To this end we intend to introduce communication controls that will allow true blocking implementations of “send” and “recv” regardless of the communication backend library used (RCCE, e.g., provides busy-wait loops for blocking). Such an implementation would increase the energy efficiency of applications built using the library. Further work on the library would involve separating the algorithmic skeletons code from the communication subsystem so that it can be retargeted to other processors. This would essentially require sand-boxing the RCCE calls behind well-structured APIs that can allow a different communication library to be plugged in.

Further, we would like to investigate the performance characteristics of all-to-all MCPSC on multicore and many-core processors with very large datasets. Experimenting with such large datasets, like the one used in [56] with more than 6000 domains, is likely to provide higher granularity and assist in more accurate assessment of the impact of different load balancing schemes in terms of speedup delivered. Additionally, cluster analysis on the results of such a large dataset will provide a better measure of the biological relevance of clusters generated by MCPSC. A dataset of that size would generate more than 3.2 million pairwise comparison tasks per PSC method and keeping the number of methods to 3 (as is the case in this work) would result in nearly 10 million tasks. Due to these large numbers, processing such a dataset specifically on a many-core processor would be feasible only with a larger NoC that is one which supports more memory and uses cores of newer architectures than the SCC. Showing that such a large computation can be completed in reasonable time would however further justify the drive towards leveraging parallel processing capabilities of multicore and many-core processors.

## Supplementary Material

The Supplementary Material contains details of hierarchical clustering for all the PSC methods used in this work. Additionally, files containing pairwise PSC scores generated for all datasets using all PSC methods are included. Finally, the sources used in this work are also included.

## Figures and Tables

**Algorithm 1:**
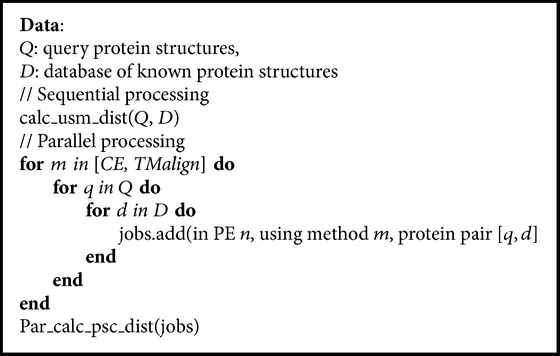
A pseudo-code of the MCPSC computation implemented in this work. The USM jobs are processed sequentially, while CE and TMalign pairwise structure comparison jobs are executed in parallel. Task *par_calc_psc_dist *assigns each PSC job to a free PE (node in a many-core and thread in multicore). If *n *is specified, the job's assignment is fixed; otherwise the next free PE available is employed.

**Table 1:** Salient features of the SCC Chip by Intel.

Core architecture	6 × 4 mesh, 2 Pentium P54c (×86) cores per tile

Local cache	256 KB L2 Cache, 16 KB shared MPB per tile

Main memory	4 iMCs, 16–64 GB total memory

**Table 2:** Basic statistics of the domains in the datasets used in this work. The table includes the number of SCOP families, superfamilies (SpFams), and folds as well as the total number of domains (No.), the Minimum (Min), Maximum (Max), Median, and Mean and Standard Deviation (Std) of the domain lengths in each dataset.

Dataset	No.	Domain lengths					SCOP classifications		
		Min	Max	Median	Mean	Std	Families	SpFams	Folds
Skolnick [57]	33	97	255	158	167.7	62.7	5	5	5
Chew-Kedem [58]	34	90	497	147	179.5	100.1	10	9	9
Fischer [59]	68	62	581	181	220.6	125.6	56	44	40
Rost-Sander [60]	114	21	753	167	193.2	123.4	93	85	71
Lancia [61]	269	64	72	68	67.9	2.4	79	72	57
Proteus [62]	277	64	728	239	247.6	116.7	53	47	41

**Table 3:** Time required for the baseline all-to-all PSC task using the TMalign, CE, and USM PSC methods on the PC and a single core of the SCC. The table also shows the time required for the all-to-all MCPSC task (where all three PSC methods are used). All times are in seconds.

Method	Ported software on PC				Ported software on SCC 1 core			
	CK34		RS119		CK34		RS119	
	Load	Processing	Load	Processing	Load	Processing	Load	Processing
TMalign	0.01	127	0.05	1725	0.30	2514	1	33452
CE	0.80	374	3	6459	14	7152	50	132205
USM	0.01	0.60	0.04	7	0.70	30	2	345
All	0.80	502	3	8191	15	9697	53	166000

**Table 4:** Sum of squares of residuals after curve fitting for the different PSC methods. The table shows that the sum of residuals is lower with quadratic fit (square) as compared to linear fit. All values are multiplied with 1*e* + 06.

PSC method	Residuals (sum of lengths)				Residuals (product of lengths)			
	CK34		RS119		CK34		RS119	
	Linear	Square	Linear	Square	Linear	Square	Linear	Square
TMalign	4	1	100	90	1	1	40	30
CE	200	100	4000	1000	100	50	2000	800

**Table 5:** Comparison of speedup and efficiency achieved by the load balancing schemes in processing the all-to-all MCPSC task vis-a-vie the single core (SCC) processing times. The efficiency is calculated assuming 47 processing elements (one PE serves as master). All times are in seconds.

Load balancing scheme	Dataset CK34			Dataset RS119		
	Time	Speedup	Efficiency	Time	Speedup	Efficiency
Serial (1 SCC core)	9698	—	—	166000	—	—
Random	479	20	0.43	4765	35	0.74
Greedy partitioning (sum)	409	24	0.50	4300	39	0.82
Greedy partitioning (product)	396	28	0.59	4433	38	0.80
Round-robin (sum sorted)	239	41	0.86	3930	42	0.90
Round-robin (product sorted)	238	41	0.86	3930	42	0.90

**Table 6:** Speedup, efficiency, and throughput in pairwise-PSC tasks per second for performing MCPSC on an Intel Core i7 multicore CPU using the CK34 and RS119 datasets.

Threads	Dataset CK34			Dataset RS119		
	Speedup	Efficiency	Throughput	Speedup	Efficiency	Throughput
1	1.00	1.00	4.92	1.00	1.00	3.49
2	1.88	0.94	9.27	1.46	0.73	5.09
3	2.04	0.68	10.01	1.98	0.66	6.90
4	2.54	0.63	12.47	2.60	0.65	9.08
5	2.70	0.54	13.25	2.70	0.54	9.42
6	2.71	0.45	13.32	2.71	0.45	9.45
7	2.74	0.39	13.47	2.69	0.38	9.38
8	2.64	0.33	12.98	2.67	0.33	9.31

**Table 7:** Comparison of the SCC and i7 CPU in terms of throughput delivered on all-to-all MCPSC.

Dataset	Pairs	SCC throughput	i7 throughput	Ratio
Skolnick	1089	6.05	27.92	4.62
Chew-Kedem	1156	5.03	13.29	2.64
Fischer	4624	1.98	9.37	4.74
Rost-Sander	12996	3.30	9.38	2.85
Lancia	72361	—	222.65	—
Proteus	76729	—	6.77	—
