# Supplementary material for: Efficient Multicriteria Protein Structure Comparison on Modern Processor Architectures
Source: Biomed Res Int. 2015 Oct 28;2015:563674. doi: 10.1155/2015/563674 (PMC4641208; doi:10.1155/2015/563674)
Supplement: Supplementary file 1 — The Supplementary Material contains details of hierarchical clustering for all the PSC methods used in this work. Additionally, files containing pairwise PSC scores generated for all datasets using all PSC methods are included. Finally, the sources used in this work are also included. [file 563674.f1.zip › 563674.f1/supplementary_material.pdf]

Supplementary Material

Efficient multi-criteria protein structure comparison on modern processor architectures

Anuj Sharma and Elias S. Manolakos

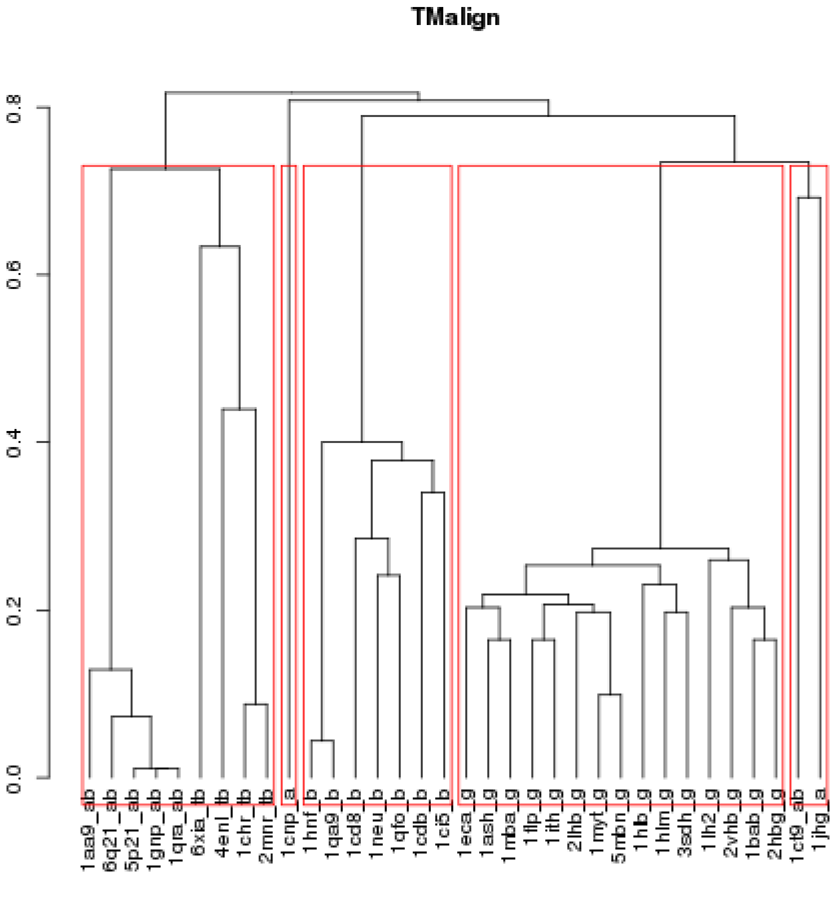

Figure S1: Hierarchical clustering result using the Chew-Kedem dataset and TM-score as distance metric between domains. Each box represents a cluster and the domains belonging to it. Average linkage was used.

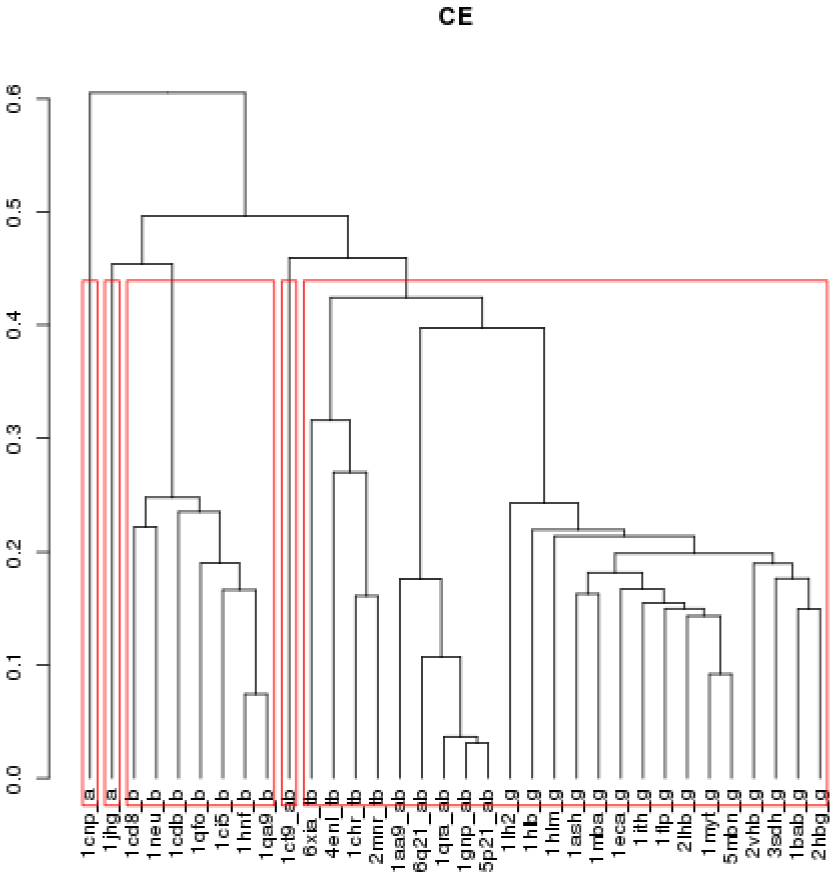

Figure S2: Hierarchical clustering result using the Chew-Kedem dataset and Root Mean Square Deviation (RMSD) from CE as distance metric between domains. Each box represents a cluster and the domains belonging to it. Average linkage was used.

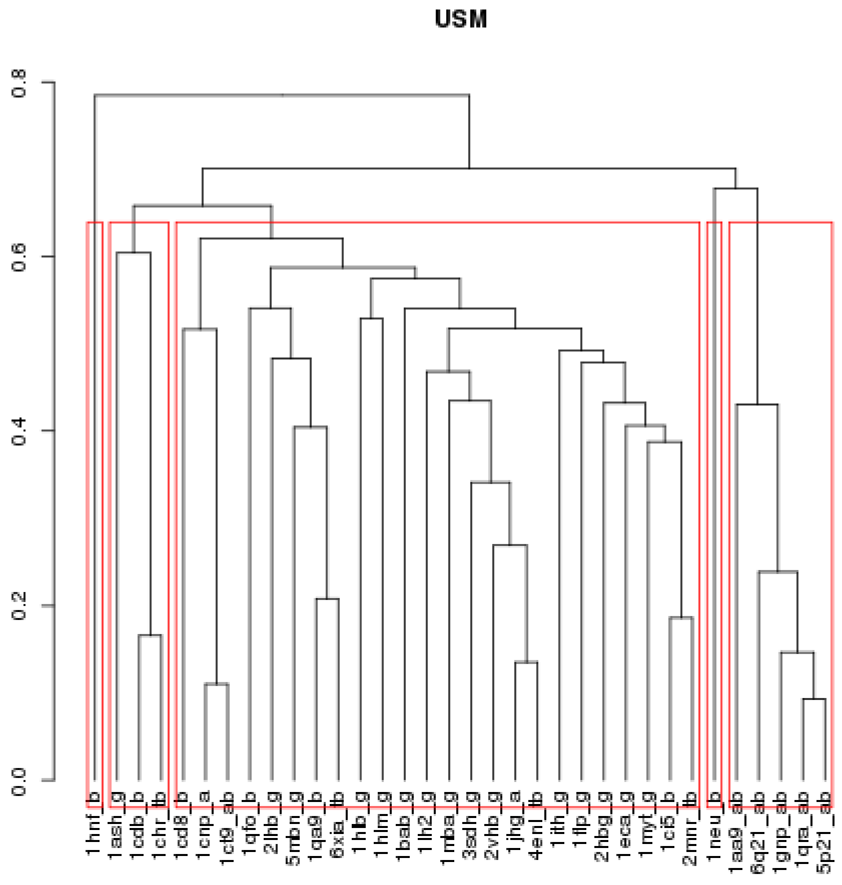

Figure S3: Hierarchical clustering result using the Chew-Kedem dataset and Universal Similarity Metric (USM) as distance metric between domains. Each box represents a cluster and the domains belonging to it. Average linkage was used.
